# Supplementary material for: Differential Co-Abundance Network Analyses for Microbiome Data Adjusted for Clinical Covariates Using Jackknife Pseudo-Values
Source: ArXiv. 2023 Mar 23:arXiv:2303.13702v1. Preprint. [Version 1] (PMC10055480)
Supplement: 1 [file NIHPP2303.13702V1-supplement-1.pdf]

Appendix A Supplementary Table

Table S1 Comparison of computational time of SOHPIE-DNA with that of NetCoMi and MDiNE. Multivariable setting is depicted for the illustrative purpose. For each sample size, the minimum and maximum computational times among various combinations of effect sizes are selected. Random network is generated at each simulation replicate.

		Minimum-Maximum Time (in hours)		
p	n	SOHPIE	NetCoMi	MDiNE
20	20	0.92-1.98	0.47-0.58	9.00-13.68
	50	2.12-3.42	0.52-0.80	8.17-15.95
	200	9.57-13.85	0.43-0.52	11.00-45.00
	500	28.40-50.40	0.57-0.80	11.00-50.63
40	20	2.13-3.15	0.73-1.52	14.67-32.63
	50	3.47-6.32	0.8-0.93	37.62-58.00
	200	14.97-19.62	0.92-1.43	180.65-320.28
	500	50.73-80.05	1.22-2.78	455.47-496.80
